# A Case of Balo Concentric Sclerosis: A Multiple Sclerosis Mimic

**DOI:** 10.7759/cureus.67076

**Published:** 2024-08-17

**Authors:** Mary Therese Thomas, Molly E Boyko, Rachel Hemsath, Kedareeshwar S Arukala, Victor Collier

**Affiliations:** 1 Internal Medicine, Grand Strand Medical Center, Myrtle Beach, USA; 2 Internal Medicine, Edward Via College of Osteopathic Medicine, Spartanburg, USA

**Keywords:** concentric demyelination, stereotactic biopsy, demyelinating disorders, s: multiple sclerosis, balo concentric sclerosis (bcs)

## Abstract

Balo Concentric Sclerosis (BCS) is a rare neurological demyelinating disorder similar to Multiple Sclerosis. Both present with progressive neurological debility but differences on brain imaging help with distinction. The lack of prevalence and general diagnostic information about BCS makes it an underdiagnosed disease which can sometimes delay treatment. This case of BCS was initially treated as an infectious brain mass, leading to unnecessary interventions. Early recognition and differentiation from other neurological conditions are crucial for appropriate management and prognosis. We hope that by presenting this case, we can aid in creating diagnostic criteria and promote awareness of this chronic debilitating disease.

## Introduction

As the most common demyelinating disorder, approximately 1.8 million people are affected by multiple sclerosis (MS) [1, 2]. Risk factors for developing MS include being a middle-aged white female, having a family history of the disease, Epstein-Barr Virus (EBV) exposure, and living further from the equator [1, 2]. Common symptoms include deteriorating vision and weakness of the extremities which may affect a patient's ability to walk and carry out other activities of daily living [1]. The etiology of MS is unclear but the leading hypothesis suggests autoimmune activity on the central nervous system (CNS) as a result of cross-reactivity involving unknown antigens that trigger CD4 proinflammatory T-cell response [3]. This causes chronic inflammation that leads to demyelination [3]. 

This disease is a diagnosis of exclusion and involves careful history-taking and physical examination. The McDonald Criteria defines the diagnosis as a series of lesions and symptoms that are disseminated in space and time, meaning there is neurological demyelination that has occurred over time and in multiple areas of the CNS [3, 4]. Cerebral Spinal Fluid (CSF) studies show oligoclonal bands and increased total IgG with elevated protein levels and mononuclear leukocytosis [3]. The Barkhof Criteria aids in radiological diagnosis and requires three of the following be present for a diagnosis of MS: (1) One or more gadolinium-enhancing lesions or nine lesions on T2-weighted MRI, (2) three or more periventricular lesions, (3) one or more juxtacortical lesion, and/or (4) one or more infratentorial lesions [5]. Treatment is focused on reducing the severity of symptoms and preventing relapses as there is no definitive cure. 

Balo Concentric Sclerosis (BCS) is an extremely rare subtype of MS, with little known of its incidence or prevalence [6]. BCS shares clinical and pathologic similarities with MS, namely, progressive demyelination [7]. Cases of BCS have an acute onset and progression to significant disability or even rapid deterioration leading to death over the course of weeks to months [6-8]. The hallmark feature on MRI is a concentric pattern of alternating rings of myelinated and unmyelinated tissue [6-8]. The underlying pathophysiology of this unique concentric pattern is unknown; however, it is said to resemble a hypoxic-like response to demyelination [6, 8]. Similarly to MS, oligoclonal bands on CSF analysis are not always seen [8]. Treatment with corticosteroids has been shown to improve daily functioning in those with BCS [3, 8]. Below, we present a case of BCS that was initially misdiagnosed and ultimately led to delayed diagnosis and treatment.

## Case presentation

A 48-year-old woman with a past medical history of hypertension presented with progressive left upper and lower extremity weakness and numbness for one year and an acute worsening of vision in the left eye for one month. She described experiencing numerous blind spots in her left eye, which hindered her ability to work. She endorsed headaches that woke her from sleep, along with nausea and vomiting. She also experienced sporadic contractures and tingling paresthesias affecting her left foot and hand which waxed and waned over the year prior to presentation. She denied any other associated symptoms and a review of the systems was otherwise negative. Although she was originally from Micronesia, she denied recent international travel and had been living in the same state for the past 30 years. She also denied sick contacts, recent history of head trauma, past surgeries, and hospitalizations. 

**Figure 1:**
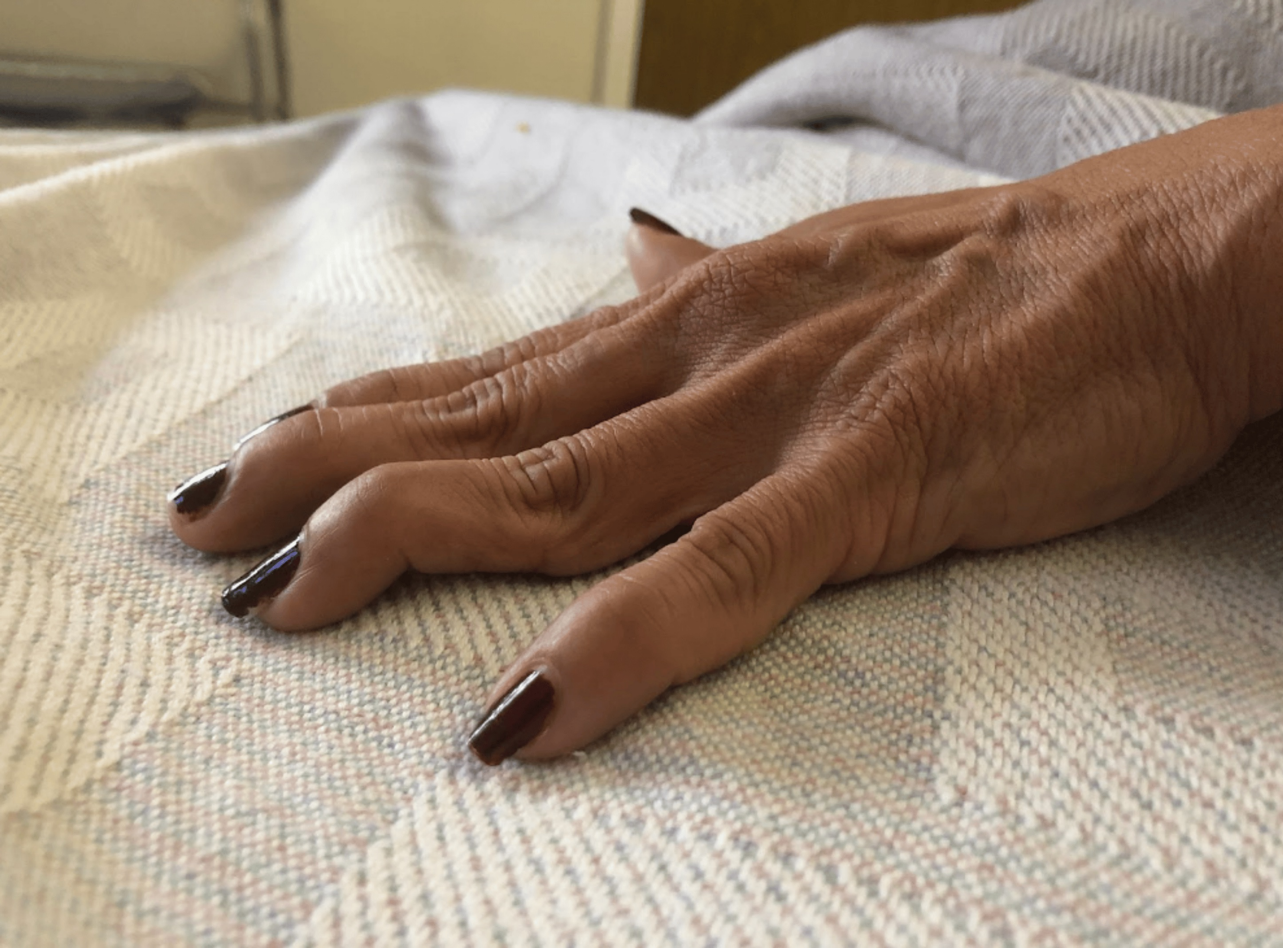
The patient's left hand contracture.

On a physical exam, the patient was alert and oriented to person, place, and time and resting comfortably in bed. A left cranial nerve II palsy was appreciated, as the patient had multiple scotomas obstructing her vision. Her right upper and lower extremity strength was 5/5 and her left upper and lower extremity strength was 3/5 with diminished grip strength and active contractures (Figure 1). Her left patellar reflex exam showed hyperreflexia without clonus. Her labs were grossly unremarkable, except for the erythrocyte sedimentation rate (ESR), which was 26 mm/hour, and lactate dehydrogenase (LDH), which was 259 units/L.

**Figure 2:**
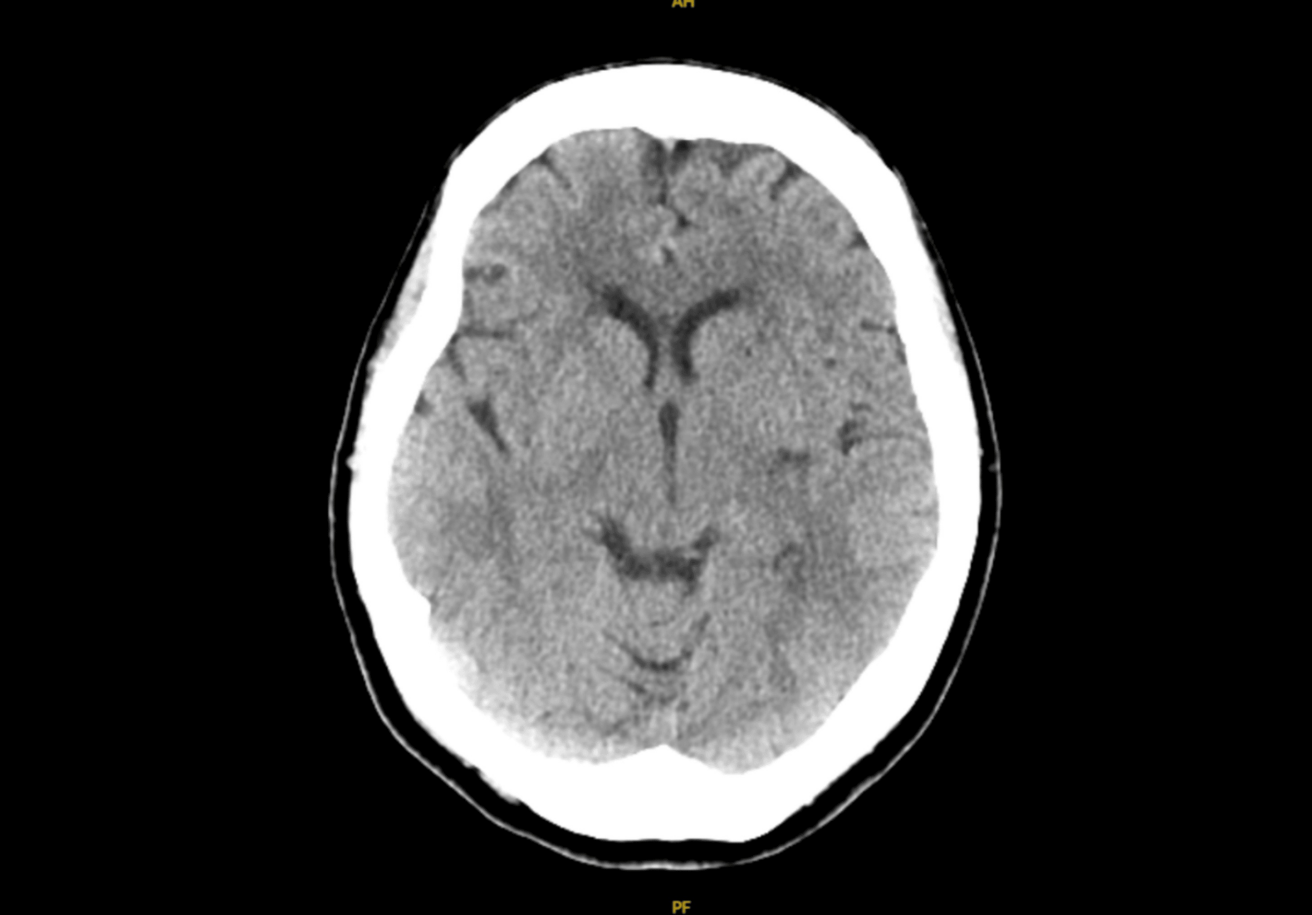
Initial CT without contrast of transverse view with age-indeterminate right frontal periventricular hypodensity.

**Figure 3:**
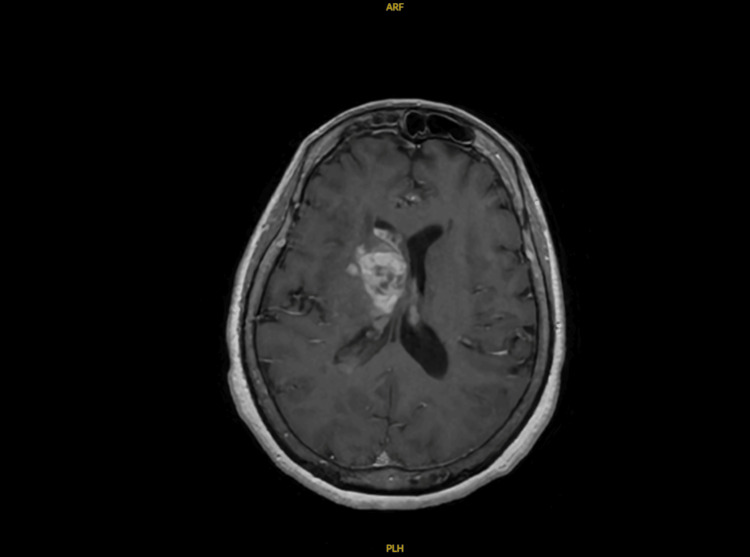
MRI T1 transverse stealth image showing a pattern of alternating hypointense-isointense and hyperintense rings in the right periventricular region, originally thought to be a cerebral abscess.

**Figure 4:**
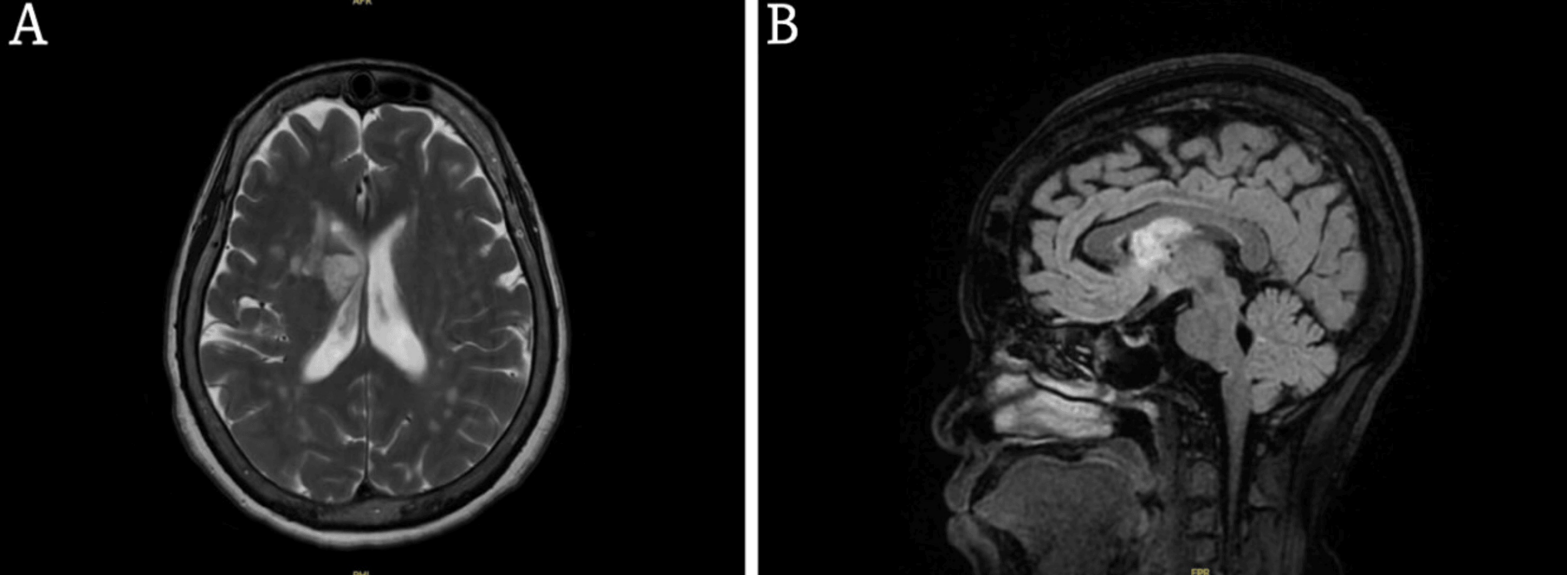
A and B. MRI T2 transverse and sagittal views showing a lesion centered within the right lentiform nucleus with associated multifocal peripheral enhancement.

**Figure 5:**
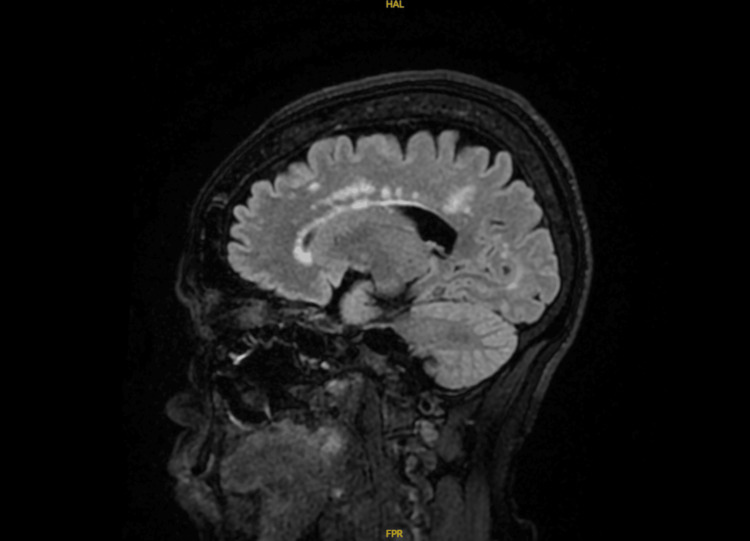
MRI T2 sagittal view showing periventricular white matter lesions.

Her initial head CT described an age-indeterminate right frontal periventricular hypodensity (Figure 2). MRI of the brain was concerning for cerebral abscess with ventricular extension and meningitis (Figures 3, 4A, 4B, 5). Neurosurgery and Infectious Disease were consulted and recommended a lumbar puncture with cerebrospinal fluid (CSF) analysis and brain biopsy of the suspected abscess. The CSF had a xanthochromic, turbid appearance and the analysis showed a WBC count of 365 cells/mm3, RBC count of 78743 cells/mm3, lymphocytes 9%, polymorphonuclear cells 90%, glucose 89 mg/dL, protein 539 mg/dL. Unfortunately, oligoclonal band studies were unable to be completed because the sample hemolyzed before processing. 

On hospital day five, she underwent a stereotactic biopsy of the right basal ganglia lesion. No purulence or fluid collections were appreciated. Samples of a blood clot found were sent for culture and pathology. Coccidioides antibody, cryptococcus antigen, and interferon-gamma testing returned negative. All cultures for the tissue biopsy returned negative as well. An echocardiogram did not show valvular abnormalities concerning for endocarditis.

Due to the lack of improvement in the patient's condition and the inconclusive results from the diagnostic tests, we were compelled to reassess our initial evidence and consider alternative explanations for the patient's symptoms. On hospital day 10, the radiologists reviewed initial imaging and suggested a possibility that this could be a demyelinating disorder, but it was irregular and inconsistent with MS. Research revealed images and reports of other patients like ours who had been diagnosed with BCS [4, 7]. Given the close similarities, it was decided to treat our patient with one gram IV methylprednisolone for three days. After a single dose, she had significant improvement in her contractures. Over the three-day course of therapy, she had near complete resolution of her weakness and was able to be discharged.

## Discussion

The diagnostic criteria for Balo Concentric Sclerosis (BCS) remains inadequately defined within the medical literature, largely owing to its exceedingly rare occurrence. Previous documentation of BCS cases has described a spectrum of diagnostic approaches. Some suggest biopsies of the lesions, while others opt for a more conservative route, trialing treatment regimens with steroids and monitoring for symptomatic improvement [7]. 

Due to this patient’s presentation and the unusual nature of her imaging, the differential diagnosis initially included MS and brain abscess. Evaluation of CSF displayed an increased protein count but the sample was hemolyzed secondary to a traumatic tap, which prevented evaluation for the presence of oligoclonal bands. Although CSF may aid in diagnosis, it is not a requirement due to the varied presence of oligoclonal bands seen in other cases of BCS throughout the literature [8]. With the patient remaining afebrile with no leukocytosis, all cultures returning negative, and a lack of improvement in the patient’s clinical status with empiric antibiotics, infectious causes were ruled out. Improvement of symptoms after the introduction of the steroid regimen supported the diagnosis of a demyelinating disease and a clinical picture consistent with BCS.

Due to the rare prevalence, high risk of misdiagnosis, and potentially severe impact on patients’ health, it is crucial to establish clearly defined diagnostic criteria for BCS. We propose that the diagnostic criteria for BCS should include the presence of classic ring-like lesions on MRI, characteristic clinical findings indicative of demyelination, and cerebrospinal fluid (CSF) analysis that excludes other potential causes. Additionally, evidence of clinical improvement with steroid treatment should be considered to support the diagnosis. These criteria are not only essential to differentiate BCS from other conditions such as MS, but also to prevent unnecessary medical interventions and delayed diagnosis. By emphasizing the significance of accurate diagnosis and tailored management approaches, we hope that healthcare professionals can ensure optimal care for patients with BCS, with the ultimate goal of improving their outcomes and quality of life.

## Conclusions

When evaluating a patient with suspected MS, obtaining CSF analysis and brain MRI is crucial in aiding diagnosis. However, if imaging displays concentric lesions suggestive of possible abscesses and there is a lack of suspicion for infectious etiology, we suggest initiation of IV steroids in consideration of the diagnosis of BCS. If there is no improvement in neurological symptoms, additional diagnostic evaluation should be undertaken to exclude other potential causes, such as tumors. This case represents the significance of including BCS as part of the differential diagnosis when evaluating a patient presenting with clinical features resembling MS in conjunction with a concentric-like mass on brain MRI.
